# Parameter inversion of a polydisperse system in small-angle scattering

**DOI:** 10.1107/S1600576722006379

**Published:** 2022-08-01

**Authors:** Kuangdai Leng, Stephen King, Tim Snow, Sarah Rogers, Anders Markvardsen, Satheesh Maheswaran, Jeyan Thiyagalingam

**Affiliations:** a Scientific Computing Department, STFC, Rutherford Appleton Laboratory, Didcot OX11 0QX, United Kingdom; b ISIS Neutron and Muon Source, STFC, Rutherford Appleton Laboratory, Didcot OX11 0QX, United Kingdom; c Diamond Light Source, Rutherford Appleton Laboratory, Didcot OX11 0QX, United Kingdom; Argonne National Laboratory, USA

**Keywords:** small-angle scattering, polydispersity, inversion, neutron scattering, X-ray scattering, nonlinear programming

## Abstract

A general method to invert parameter distributions of a polydisperse system using data acquired from a small-angle scattering (SAS) experiment is presented. The forward problem, *i.e.* calculating the scattering intensity given the distributions of any causal parameters of a theoretical model, is generalized as a multi-linear map, characterized by a high-dimensional Green tensor that represents the complete scattering physics. The inverse problem, *i.e.* finding the maximum-likelihood estimation of the parameter distributions (in free form) given the scattering intensity (either a curve or an image) acquired from an experiment, is formulated as a constrained nonlinear programming (NLP) problem. This NLP problem is solved with high accuracy and efficiency via several theoretical and computational enhancements, such as an automatic data scaling for accuracy preservation and GPU acceleration for large-scale multi-parameter systems. Six numerical examples are presented, including both synthetic tests and solutions to real neutron and X-ray data sets, where the method is compared with several existing methods in terms of their generality, accuracy and computational cost. These examples show that SAS inversion is subject to a high degree of non-uniqueness of solution or structural ambiguity. With an ultra-high accuracy, the method can yield a series of near-optimal solutions that fit data to different acceptable levels.

## Abstract 2

An accurate and efficient method for model- and form-free inversion of a polydisperse small-angle scattering system is presented. It supports an arbitrary number of model parameters and both 1D and 2D intensity observations.

## 1. Introduction

Small-angle scattering (SAS) is an experimental technique to probe the microstructure of a material sample by analysing the scattering pattern arising from the diffraction of incident radiation observed at small angles of emergence. As a stochastic approach, SAS can deliver statistically significant information about the shape, size, orientation and contrast of inhomogeneities from nano- to micrometre scales. Commonly used radiation sources include X-rays (SAXS, for a structural scale from 1 to 100 nm), neutrons (SANS, also from 1 to 100 nm) and light (SALS, from 100 nm to 1 mm). See Guinier & Fournet (1955) ▸, Feigin & Svergun (1987) ▸, Brumberger (2013) ▸, Lombardo *et al.* (2020) ▸ and Jeffries *et al.* (2021) ▸ for detailed overviews on SAS experimentation, data analysis and applications.

Since Lord Rayleigh described the scattering amplitude of a uniform sphere in the early 1900s (Rayleigh, 1914 ▸), an abundance of theoretical SAS models have been developed based on deterministic or stochastic wave-scattering theory. The aim of SAS data analysis can be summarized as being to determine a theoretical model that best explains the observed scattering intensity. This task can be roughly divided into two steps: model-type selection and parameter inversion.

In model-type selection, one attempts to classify the observed data under a correct model type. The solution is mostly empirical, facilitated by one’s past experience and *a priori* knowledge about the test sample. Providing a way of gathering ‘experience’ and ‘knowledge’ on a computer, machine learning has recently been employed to solve such classification problems, *e.g.* by Franke *et al.* (2018) ▸, Archibald *et al.* (2020) ▸, Do *et al.* (2020) ▸, Ikemoto *et al.* (2020) ▸ and Tomaszewski *et al.* (2021) ▸. In these studies, an end-to-end machine-learning model (either classical or a deep neural network) is trained with synthetic data generated by surrogate modelling; the trained model can then be used to classify experimental data within the regimes of the training set.

This article is concerned with the second task, parameter inversion, *i.e.* finding the best data-fitting parameters of a selected theoretical model. Depending on whether the parameters are scalar valued or distributional, we are dealing with a monodisperse or a polydisperse system, respectively. Polydispersity is naturally implied in the context of parameter inversion, as a perfect monodisperse system can be trivially optimized by a brute-force search. Technically, we can categorize the existing methods for SAS inversion into three kinds: (i) physics driven, (ii) inversion driven, and (iii) data driven or machine-learning based.

The physics-driven methods refer to those proposed in the earlier days that focus on mathematical explorations (particularly functional approximations) of the scattering physics. Some representative examples include the indirect Fourier transformation (Glatter, 1977 ▸; Moore, 1980 ▸; Hansen & Pedersen, 1991 ▸; Svergun, 1991 ▸; Brunner-Popela & Glatter, 1997 ▸; Weyerich *et al.*, 1999 ▸), direct structural analysis (Glatter, 1988 ▸; Mittelbach & Glatter, 1998 ▸), the Fedorova–Schmidt analytical method for dilute systems (Fedorova & Schmidt, 1978 ▸; Botet & Cabane, 2012 ▸; Ciccariello, 2014 ▸), and the maximum entropy method or MaxEnt (Potton *et al.*, 1988*a* ▸,*b* ▸). These methods are mostly aimed at size-distribution inversion, while a few are also available for shape and orientation determination. Some of them are still in active use, as facilitated by their visual implementations in software packages such as *SASfit* (Breßler *et al.*, 2015 ▸), *ATSAS* (Manalastas-Cantos *et al.*, 2021 ▸), *Irena* (Ilavsky & Jemian, 2009 ▸) and *GSAS-II* (Toby & Von Dreele, 2013 ▸). They also clarify some fundamental questions in SAS data analysis, such as particle interaction in a high-concentration system (Brunner-Popela & Glatter, 1997 ▸; Weyerich *et al.*, 1999 ▸). Nevertheless, relying on the scattering physics, these methods are mostly model based, *i.e.* applicable to a certain model (such as polydisperse spheres) or data type (such as 1D intensity curves). Meanwhile, their recent development towards more complex models (such as coupled size and orientation inversion) and data types (such as 2D intensity images) has notably slowed down, with attention shifting to model-free methods that utilize state-of-the-art general-purpose optimization techniques.

The inversion-driven methods are those emphasizing a physics-independent formulation of the inverse problem. Disentangling physics (or forward modelling) from inversion benefits both developers and users. As a developer, one can focus on solving one inverse problem with modern optimization techniques while implementing all kinds of models in a unified manner; while as a user, one no longer relies on some abstruse theory to understand and use these methods. Two community software packages are of this kind: *SasView* (Doucet *et al.*, 2021 ▸) and *McSAS* (Bressler *et al.*, 2015 ▸). *SasView* is built on a comprehensive Python library (*SasModels*) for SAS modelling and inversion. It solves the inverse problem by nonlinear programming (NLP), supporting both gradient-based and non-gradient optimization techniques. However, *SasView* requires the parameter distributions to take certain functional forms, such as Gaussian, log-normal and their combinations, whereby only a handful of variables are optimized (*e.g.* the mean and variance of a Gaussian). Such a restriction significantly reduces the scale of the inverse problem compared with free-form inversion, but at the cost of its data-fitting ability and ease of use (as users must correctly guess the functional forms). *McSAS* is a Python program used to invert the parameter distributions in free form by means of Monte Carlo sampling. Given infinite time, the Monte Carlo method can deliver the true posterior distributions of the variables. However, it suffers from a search space (and thus a computational cost) that quickly explodes as the number of variables grows. Furthernore, even given a long search time, the Monte Carlo method is unlikely to find the optimal solution without being guided by any gradient information. These general pitfalls limit the computational performance and accuracy of *McSAS*.

The data-driven methods are those based on machine-learning techniques. Regarding SAS inversion as a high-dimensional nonlinear regression problem, one can train a supervised model with its input and output being the scattering intensity and the model parameters, respectively, using synthetic data generated by surrogate modelling. Such a workflow has been adopted in a few recent studies (Archibald *et al.*, 2020 ▸; Demerdash *et al.*, 2019 ▸; He *et al.*, 2020 ▸; Van Herck *et al.*, 2021 ▸). Clearly, a supervised learning-based solution is highly problem specific, not only model based but also restricted to a finite sub-parameter space from which the training set is sampled. This sub-parameter space must cover the real data of interest but cannot grow very large, to avoid an exploding training set. Though lacking some generality, machine learning is still a promising tool for problem solving in SAS experimentation and data analysis (Chen *et al.*, 2021 ▸).

In this article, we describe our new method for SAS parameter inversion, which belongs to the inversion-driven kind. Our formulation of the inverse problem is physics independent, covering theoretical models with an arbitrary number of polydisperse parameters and both 1D and 2D intensity observations. Employing a versatile trust-region method as the underlying NLP solver, we simultaneously optimize all the polydisperse parameters in free form, achieving high accuracy and efficiency based on a series of theoretical and computational enhancements.

Our method has been implemented as an open-source Python library called *FFSAS* (https://github.com/stfc-sciml/ffsas, including the code and data to reproduce all the figures mentioned in *Examples* [Sec sec3]) (FF standing for free form). After describing our method, we will conduct synthetic tests and solutions to real data sets acquired from X-ray and neutron experiments, comparing *FFSAS* with *Irena* (Ilavsky & Jemian, 2009 ▸), *SasView* (Doucet *et al.*, 2021 ▸) and *McSAS* (Bressler *et al.*, 2015 ▸) from many aspects.

## 2. Methods

### 2.1. Forward problem

Our forward problem is to calculate the scattering intensity given a theoretical SAS model and its parameter distributions. We generalize this problem as a high-dimensional multi-linear map so as to benefit from a physics-independent formulation of the inverse problem.

Consider a SAS model with *N* polydisperse parameters: ^1^ *p*, ^2^ *p*,…, ^*N*^ *p*. For instance, *N* = 1 for spheres (the only parameter being radius) and *N* = 4 for cylinders (the four parameters being radius, length and two angles of orientation with respect to the beam). We discretize the parameter space of ^*k*^ *p* by a vector of size *n*_*k*_, 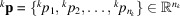 . Let 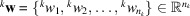 be the density distribution of ^*k*^ *p*, that is, ^*k*^ *w*_*i*_ being the number fraction of ^*k*^ *p*_*i*_, subject to ^*k*^ *w*_*i*_ ≥ 0 and 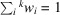 . The *N* density distributions, ^*k*^ **w**, are input for the forward problem and output for the inverse problem.

The scattering intensity *I* is a function of *M* scattering vectors, that is, 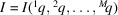 . Discretizing ^*k*^ *q* by vectors 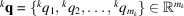 , we obtain a discretized intensity as an *M*th rank tensor, 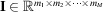 with 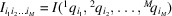 . In real SAS experiments, *M* can be 1 or 2, corresponding to **I** being a 1D curve or a 2D image, respectively.

Having the above definitions, the forward problem can be formulated as the following multi-linear map (Einstein summation convention is not adopted in this article): 

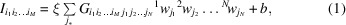

where ξ and *b* are two scalars, and 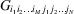 is a tensor of rank (*M* + *N*). Physically, **G** represents the square of the scattering amplitude, normally denoted by *F* ^2^. For a dilute system, 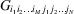 equates to the *F* ^2^ produced by a monodisperse system with parameters 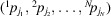 and observed at point 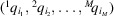 in the *q* space, also known as the form factor. Because **G** defines the local behaviour of a linear reaction system, we call it the Green tensor of polydispersity. Scalar ξ is the total volume fraction of inhomogeneities divided by the average volume of inhomogeneities and scalar *b* is the source background. In the inverse problem, we will solve ^*k*^ **w**, ξ and *b* as variables, assuming that **G** provides a complete representation of the scattering physics.

Take polydisperse spheres with 1D data, for example: we have *M* = *N* = 1, with radius *r* being the only model parameter. Green’s tensor for a dilute system (Rayleigh, 1914 ▸) can be shown as (with ^1^ *p* and ^1^ *q* written as *r* and *q*, respectively) 

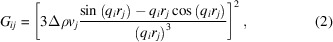

where *v*_*j*_ is the volume of a sphere, 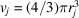 , and Δρ is the difference between the scattering-length density of the spherical inclusions and that of the matrix. When the contrast Δρ^2^ is unknown (as is often the case in practice), one can ‘merge’ it into ξ for inversion by computing **G** with Δρ = 1; in that case, the contrast and the total volume fraction form a pair of non-separable trade-offs via their product ξ.

Our forward formulation (and thus the subsequent inverse formulation) can cover any physical or experimental effects conveyable by the Green tensor. In particular, we emphasize the following four effects:

(*a*) Particle interaction. In a high-concentration system, the multi-scattering effects among particles become unignorable. According to one of the early established decoupling theories, such multi-scattering effects can be built into equation (1) via certain analytical corrections of the **G** determined by local monodispersity. The most commonly used theory is the ‘*G* = *P* *S*’ factorization (Brunner-Popela & Glatter, 1997 ▸; Weyerich *et al.*, 1999 ▸), where *P* is the form factor and *S* is the structure factor. For a high-concentration system, **G** may no longer be a constant but involve a few extra variables to be inverted jointly with ^*k*^ **w**, ξ and *b*, such as the effective size and volume fraction of the inclusions.

(*b*) Resolution functions. To compensate for the experimental effect of *q*-resolution smearing, one can apply a resolution function to correct the theoretical intensity prediction (Pedersen *et al.*, 1990 ▸). Obviously, any correction of the intensity prediction can be directly integrated into **G**. In practice, a linear correction is usually applied: assuming *M* = 1 for simplicity, 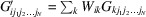 , where the coefficients *W*_*ik*_ are determined by the **q** vector (and its variance if available) in several ways; see the *SasView* (Doucet *et al.*, 2021 ▸) documentation for details.

(*c*) Contrast-varying systems. From an inversion viewpoint, equation (1) also covers a polydisperse system with a varying contrast because the intensity **I** simply scales with the contrast ξ. For example, given a system with two populations of spheres characterized by (ξ_*A*_, **w**_*A*_) and (ξ_*B*_, **w**_*B*_), one can always find its ‘uniform-contrast equivalence’ (ξ_*U*_, **w**_*U*_) such that ξ_*U*_ **w**_*U*_ = ξ_*A*_ **w**_*A*_ + ξ_*B*_ **w**_*B*_, where 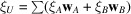 and **w**_*U*_ = (ξ_*A*_ **w**_*A*_ + ξ_*B*_ **w**_*B*_)/ξ_*U*_. In short, a uniform-contrast system can be interpreted as an infinite number of contrast-varying systems (if only comparing their induced intensities), so an inversion with multiple contrasts (ξ values) is extremely underdetermined and makes little sense. It does make sense, however, for a heterogeneous system that involves two or more forward models (*e.g.* a mixture of spheres and cylinders) because their Green tensors differ. Such heterogeneous systems are not considered in this article.

(*d*) Non-uniform background. Sometimes a non-uniform source background may be required to better fit the intensity data. For such cases, instead of having a scalar *b* in equation (1), we can write the background as a function of the scattering vectors, *i.e.* 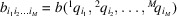 . Such a background function cannot be too expressive; otherwise, the intensity data may be fitted solely by the background without optimizing the parameter distributions. In practice, a power law is frequently used (Ilavsky & Jemian, 2009 ▸), *i.e.* 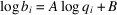 for *M* = 1, where the coefficients *A* and *B* can be given by the user or inverted jointly with ^*k*^ **w** and ξ.

### 2.2. Inverse problem

From a SAS experiment, one can observe the mean and standard deviation of the scattering intensity, *i.e.* 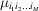 and 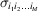 at 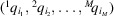 . Given a target SAS model and its parameter space ^*k*^ **p**, the Green tensor **G** can be determined. The inverse problem is to optimize ^*k*^ **w**, ξ and *b* so that 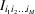 determined by equation (1) can best explain the observations, given 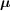 , 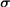 and **G** as input data.

To quantify the goodness of fit, it is natural to maximize the following likelihood function 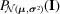 : 

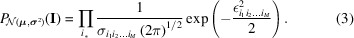

Here 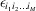 denotes the 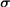 -normalized intensity misfit, 

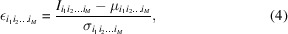

with 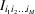 given by equation (1). The normalization by 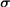 takes uncertainty of the data into account: points with larger variances will contribute less to the likelihood. It also serves the purpose of regularization: the values of **I** may span several orders of magnitude for widely ranged scattering vectors, making the absolute error 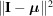 insensitive to the smaller values. When 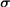 is unavailable from an experiment, one can use 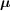 to take its place in equation (4); doing so, one assumes that the measurement error scales with the measured amplitude at a detector.

By taking the logarithm of 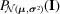 , one can show that the above maximum-likelihood problem is equivalent to minimizing the squared Frobenius norm of 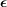 , 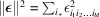 , also known as the χ^2^ error. Eventually, the inverse problem can be formulated as the following constrained NLP, here named NLP-w:

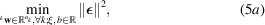

subject to

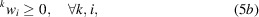




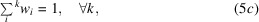

where 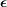 is determined by equation (4). Equation (5*a*) means that we aim to find the values of ^*k*^ **w**, ξ and *b* that minimize 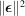 , subject to the constraints in equations (5*b*) and (5*c*) that require each ^*k*^ **w** to have non-negative elements summing to 1. The presence of a structure factor or a non-uniform background may introduce extra variables into NLP-w, which can be handled by a general optimization algorithm in the same manner as ^*k*^ **w**, ξ and *b*. The minimizer of NLP-w is called the maximum-likelihood estimator (MLE), in light of equation (3).

NLP-w is an ill-posed large-scale NLP with mixed equality and inequality constraints. To solve it with high accuracy and efficiency, we have implemented several theoretical and computational enhancements. They are all elaborated in Appendix *A* [App appa]; here we only take a quick tour. To make NLP-w solvable, we first introduce a slack variable to eliminate the inequality constraints in equation (5*b*), turning NLP-w into another NLP named NLP-s (Appendix *A*1[App appa]). Next, we introduce an automatic approach to rescale the input data for accuracy preservation (Appendix *A*2[App appa]). This makes our method highly accurate, as we will show in *Examples* [Sec sec3]. Finally, to solve NLP-s with the auto-scaled data, we use the Byrd–Omojokun trust-region method (Lalee *et al.*, 1998 ▸) implemented in *SciPy* (Virtanen *et al.*, 2020 ▸), with its computational performance boosted by two techniques: GPU-accelerated chunk computation (Appendix *A*3[App appa]) and on-the-fly dimension reduction (Appendix *A*4[App appa]). A GPU is needed only for large-scale multi-parameter problems; for a low-dimensional problem such as size-distribution inversion of polydisperse spheres (*N* = 1), even at an ultra-high resolution, our runtime is usually a few seconds on a CPU.

### 2.3. Sensitivity and uncertainty

Once the MLE is found, we can further conduct sensitivity and uncertainty analysis, both delivering important characteristics of the solution. The sensitivity can indicate which model parameters or parameter ranges are dominating the locality of the MLE, while the uncertainty shows our confidence in the MLE.

For sensitivity analysis, let **X** denote the flattened vector containing all the variables, **X** = {^*k*^ **w**, ξ, *b*} (with size 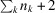 ), and let **J** and **H** denote the Jacobian and Hessian vectors, respectively, of 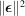 with respect to **X**, *i.e.* 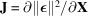 and **H** = ∂**J**/∂**X**. Let 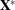 be the minimizer of NLP-w or the MLE. The normalized sensitivity at 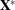 is then determined by 

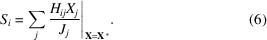




With uncertainty analysis, we aim to determine the error bar for each variable by back-propagating the observational error. For a general nonlinear problem, a Monte Carlo sampling is usually required to find the joint-posterior distribution of the variables; linearizing this joint posterior at the MLE will give a covariance matrix whose diagonal can be used as the error bars (Tarantola, 2005 ▸). However, the forward problem of SAS, equation (1), is special in that the intensity is a linear function of each ^*k*^ **w** at the MLE, which enables us to determine this linearized covariance matrix analytically.

Let 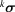 denote the standard deviation (or error bar) of ^*k*^ **w**, which can be computed using the following equation [see equation (3.56) of Tarantola (2005) ▸]: 

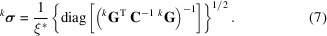

Here 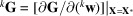 , *i.e.* the inner product of **G** with all the MLE weights except 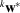 , 

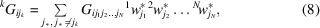

and **C** is the covariance matrix of the intensity observation, which is diagonal with 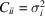 . In equation (8), the *q* dimensions 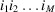 are flattened into one dimension *i*.

## 3. Examples

We implement our method as an open-source Python library named *FFSAS*. In this section, we will present six examples to demonstrate its usage and features, including three synthetic recovery tests and three real data sets acquired from a SANS or SAXS experiment. We will compare the solutions given by *FFSAS* with those by three existing software packages: *Irena* (Ilavsky & Jemian, 2009 ▸), *SasView* (Doucet *et al.*, 2021 ▸) and *McSAS* (Bressler *et al.*, 2015 ▸).

### 3.1. Benchmark: spheres with an analytical bi-model size distribution

**Figure 1:**
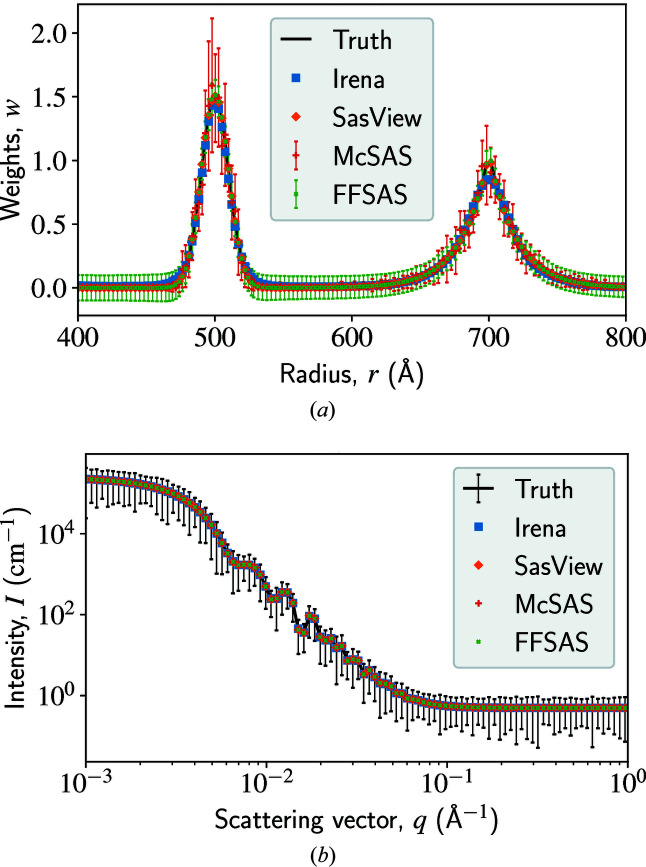
A benchmark for size-distribution inversion of polydisperse spheres. (*a*) shows the true and inverted size distributions; the truth is composed of two analytical parts, a Gaussian on the left and a Boltzmann on the right, with the radius ranging from 400 to 800 Å and discretized by 500 points. (*b*) shows the true and fitted intensity curves, with *q* ranging from 10^−3^ to 1 Å^−1^ and discretized by 200 points in logarithmic scale; we add a 20–30% error to the intensity observation (we use 3σ for the error bars in this plot). To obtain the *SasView* solution, we need to create a user-defined model combining a Gaussian and a Boltzmann distribution, and set their initial peaks close enough to the truth. For *Irena* (MaxEnt), we need to decrease the observational error to 1% to achieve an accuracy comparable to that of the other three solutions. The metrics are summarized in Table 1 ▸.

In this example, we conduct a benchmark solution for polydisperse spheres with a size distribution composed of two analytical functions, one Gaussian and one Boltzmann, as shown in Fig. 1 ▸(*a*) as the ‘Truth’. We compute the scattering intensity using this size distribution, then assume a 20–30% error at each data point to create a complete intensity observation, as shown in Fig. 1 ▸(*b*) as the ‘Truth’. Regardless of the assumed observational error, the MLE of the size distribution is always the bi-model truth. Our task is to recover the true *w*(*r*) from the true *I*(*q*) using *FFSAS* and the other three codes. More details of the problem are given in the caption of Fig. 1 ▸.

The solutions yielded by the four codes are shown in Fig. 1 ▸, with their fitting errors and computational cost given in Table 1 ▸. Generally speaking, the four solutions all deliver a good intensity fit, as shown in Fig. 1 ▸(*b*). Let us evaluate them more closely. The MaxEnt solution from *Irena* has the largest χ^2^ error, which is understandable as the objective function of MaxEnt is not exactly χ^2^ but the sum of it and another entropy term. The largest misfits occur near the two peaks of *w*(*r*). To achieve this reported accuracy, we need to decrease the assumed observational error to 1%. The *SasView* solution is more accurate in terms of both *I*(*q*) and *w*(*r*). It is the fastest solution among the four, since we have informed *SasView* that the target size distribution must contain a Gaussian and a Boltzmann, so it only needs to optimize their peak locations and widths. Similarly to *SasView*, *McSAS* achieves an intermediate-high accuracy, with some large misfits occurring near the two peaks; being sampling based, this solution is much more expensive than the others. In comparison, *FFSAS* delivers the highest-quality solution to this benchmark problem, diminishing χ^2^ to a near machine-epsilon level at a fast speed while requiring no prior information or data simplification.

As a recovery test with a simple ground truth, this example shows that *FFSAS* has the strongest data-fitting capability, owing to our algorithmic enhancements (see Appendix *A* [App appa]) that have not been considered before. However, a solution that better fits the data is not necessarily more physically sound. The reason for this is that SAS inversion is subject to a high degree of structural ambiguity, which we will visualize and discuss in later examples.

### 3.2. Spheres with a drastically varying size distribution

**Figure 2:**
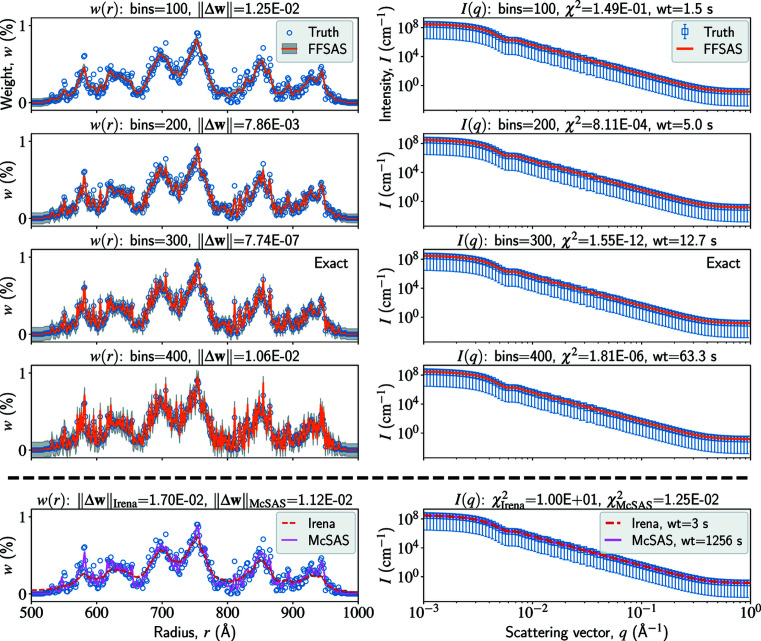
A multi-resolution synthetic test on size-distribution inversion of polydisperse spheres. The left and right columns show the radius distribution *w*(*r*) and the scattering intensity *I*(*q*), respectively. The resolutions of 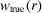 and 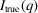 are 300 and 2000, respectively. With *FFSAS*, we use four different resolutions of the inverted radius distribution 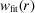 , and the results are shown in the first four rows. The last row shows the solutions from *Irena* (MaxEnt) and *McSAS*, both using 300 as the resolution of 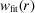 . The wt values are measured on a CPU.

Much like the previous one, this example is a recovery test for polydisperse spheres. However, here we make the problem much more challenging by using a drastically varying stochastic size distribution. The ground truth of the radius distribution, 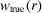 , and its induced scattering intensity, 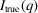 , are shown in Fig. 2 ▸. We attempt to recover 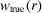 using 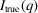 as both the mean and standard deviation of the intensity observation. Dominated by a short-wavelength large-amplitude white noise, 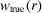 can be recovered only with a highly accurate inverse solver.

With *FFSAS*, we try four different resolutions (or bin numbers) of the inverted radius distribution 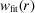 . The results are shown in Fig. 2 ▸. Let us first compare the 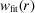 curves in the left column. Using the resolution of 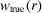 for 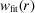 , *FFSAS* can exactly recover 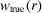 (the third row). The 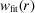 curves obtained at the lower resolutions behave well as smooth interpolations of 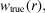 but those obtained at the higher resolutions exhibit some overshooting. Even using the resolution of 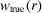 , *Irena* and *McSAS* can only yield a much smoother 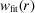 (the last row). Now we look at the intensity fit in the right column. Though the 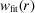 curves look quite different, their quality of intensity fits visually look the same. For example, the χ^2^ error of the *FFSAS* solution is smaller than that of the *Irena* solution by 10^12^, but their predicted 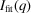 curves look similar.

The fact that distinct *w*(*r*) curves predict very similar *I*(*q*) curves indicates the ill-posedness of the inverse problem: the neighbourhood of the MLE is nearly flat (though convex), leading to a high degree of non-uniqueness of solution or structural ambiguity. This has important practical implications. First, given an intensity observation with a certain noise level, a solution closer to the MLE (or with a smaller χ^2^) could be less physically plausible because of overfitting. Regularizing the χ^2^ error with some additional constraints is one way of selecting a solution near the MLE, such as MaxEnt (Potton *et al.*, 1988*a* ▸,*b* ▸), but regularization is also a subjective non-physical choice. What we recommend is to provide a series of solutions that fit the data to different acceptable levels, from underfitting to overfitting, so that the user can select a solution on the basis of other physical or empirical considerations. However, entering the overfitting regime requires a highly accurate inverse solver, and the lower the noise level is, the more accurate the inverse solver needs to be. In this example, our intensity data are noise free, for which only *FFSAS* can approach the overfitting regime (χ^2^ ≃ 10^−12^), whereas the other codes mainly work in an underfitting regime (10^−4^ < χ^2^ < 10^0^).

### 3.3. Cylinders with four polydisperse parameters

In this example, we demonstrate the solution of a large-scale problem. Consider polydisperse cylinders with four parameters: length *l*, radius *r*, angle from cylinder axis to beam θ and rotation of cylinder axis about beam ϕ, all discretized by 40 points. The intensity observation is a 2D image, *I* = *I*(*q*_*x*_, *q*_*y*_), with *q*_*x*_ and *q*_*y*_ both discretized by 120 points. Consequently, the shape of the Green tensor is 120 × 120 × 40 × 40 × 40 × 40, occupying 295 GB of memory in double-precision floats. So far as we know, this problem cannot be solved by any of the existing codes for SAS data analysis.

**Figure 3:**
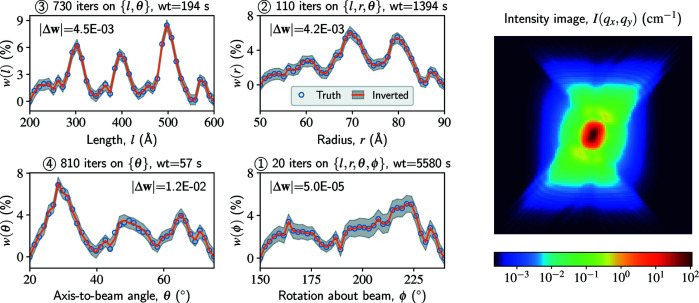
A large-scale synthetic test on size- and orientation-distribution inversion of polydisperse cylinders. The parameter distributions (truth and inverted) are shown on the left, all discretized by 40 points. The intensity image is shown on the right (truth and fitted look identical), with *q*_*x*_ and *q*_*y*_ both ranging from −1 to 1 Å^−1^ and discretized by 120 points. A preparatory solution with a low-resolution *q*_*x*_ and *q*_*y*_ (40 × 40) is first conducted to provide a good initial guess for the high-resolution inversion. During the high-resolution inversion, we monitor the parameter distributions every ten trust-region iterations and compute the L1 distance between two records to decide whether any of them have converged. The parameters converge in the sequence of ϕ, *r*, *l* and θ, as indicated by the circled number in each title; a converged parameter is fixed for further iterations. The wt values are measured on a NVIDIA Tesla V100 GPU.

We solve this problem in two steps. First, we conduct a preparatory solution with a lower-resolution *q*_*x*_ and *q*_*y*_ (*i.e.* using a decimated intensity image as the input), which can provide a good initial guess for the original problem. Next, starting from this initial guess, we conduct the high-resolution inversion with on-the-fly dimension reduction (see Appendix *A*4[App appa]). The results in Fig. 3 ▸ show that the four parameter distributions are all recovered with high accuracy. The solving process has undergone reductions of dimension in the sequence of ϕ, *r*, *l* and θ; after each reduction, a trust-region iteration becomes roughly 40 times faster. The wall-clock time (wt) is ∼2.2 h using a GPU (including the preparatory solution), which would be increased by one to two orders of magnitude without on-the-fly dimension reduction.

### 3.4. SANS from polydisperse spheres

This SANS data set is acquired from a 0.5%(*v*/*v*) charge-stabilized polystyrene latex dispersed in a 1 m*M* aqueous sodium chloride buffer made up in heavy water (Hellsing *et al.*, 2012 ▸). On the basis of a *SasView* model fit assuming polydisperse spheres, the authors reported a Gaussian distribution of 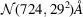 for the particle sizes. They carried out certain instrumental corrections in processing their data which, because they do not elucidate them, we have been unable to replicate here. Therefore, our results from *SasView* may slightly differ from the published ones; however, this does not hinder our purpose of method demonstration and comparison.

In this and the next example, we will use the volume-weighted density distribution, as denoted by 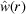 , 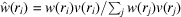 , *i.e.* the normalized volume fraction of inclusions. Compared with the number fraction *w*(*r*), 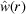 is more physically meaningful (as it approximately scales with the scattering amplitude) and is thus presented more frequently as the final outcome of size-distribution inversion. One can also directly use 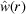 as the variable for inversion; in *FFSAS*, for example, one can do so simply by using *G*_*ij*_/*v*_*j*_ as the Green tensor, with *G*_*ij*_ given by equation (2). Whether *w*(*r*) or 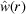 will serve better as the inverse variables depends on which of them is more regular across the radius range of interest.

**Figure 4:**
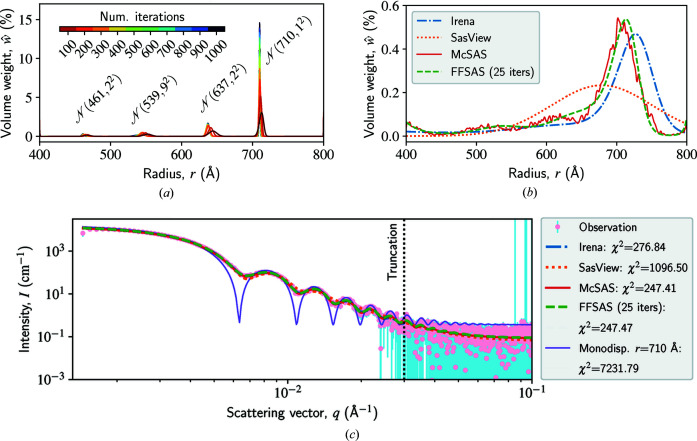
Size-distribution inversion of polydisperse spheres using a SANS data set. The intensity data contain 986 points; we cut off its noisy high-*q* end to keep 285 points for inversion. The radius *r* ranges from 400 to 800 Å, discretized by 1000 points. (*a*) shows the convergence of 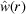 in one *FFSAS* run through the trust-region iterations; the final one suggests four populations, as annotated by their Gaussian approximations. (*b*) compares the 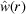 curves obtained by the four codes; for *SasView*, we use one Gaussian as the functional form. Because *Irena* (MaxEnt), *SasView* and *McSAS* all yield a flat 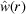 , we choose one of the early *FFSAS* solutions (after 25 iterations) for the comparison. The area under all the 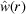 curves is 1, so the *y*-axis scale of (*b*) (dispersive or flat) is much smaller than that of (*a*) (localized or spiky). (*c*) shows the intensity observation and the *I*(*q*) curves predicted by the 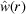 curves given in (*b*), plus one for perfect monodispersity at 710 Å as a baseline.

The intensity data and our results are shown in Fig. 4 ▸. Let us first examine the radius distributions in Figs. 4 ▸(*a*) and 4 ▸(*b*). Fig. 4 ▸(*a*) displays the convergence of 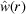 in one *FFSAS* run: as the trust-region iterations proceed, 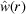 becomes increasingly more localized or spiky and finally converges to a four-population distribution dominated by 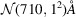 . Comparing our final 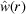 (after 1000 iterations) with the published one (Hellsing *et al.*, 2012 ▸) we see that, while both yield a mean value near 700 Å, our standard deviation (1 Å) is much smaller, which seems more consistent with the reported low dispersity of the particles. The other three minor populations (centred at 461, 539 and 637 Å) significantly improve the goodness of fit near the turning points of the intensity curve, as compared with the baseline solution of perfect monodispersity at 710 Å in Fig. 4 ▸(*c*). We cannot explain these minor populations physically, although they could result from experimental artefacts or model imperfection. Anyway, we do not claim that our solution is more physically sound than the reported one.

In Fig. 4 ▸(*b*), we compare the 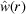 curves obtained by the four codes. Because *Irena*, *SasView* and *McSAS* all yield a highly dispersive or flat 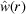 , we compare their solutions with one of the early *FFSAS* solutions (after 25 iterations). Fig. 4 ▸(*b*) shows that the *McSAS* and *FFSAS* solutions are in good agreement, while the *SasView* solution (as it is assumed to be a Gaussian) is far away from the others. Though being form free, the *Irena *and *McSAS* approaches cannot obtain any of the localized or spiky distributions seen in Fig. 4 ▸(*a*), because, once the χ^2^ error has reached some small value, they cannot keep minimizing it at a higher precision. The area under all the 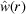 curves is 1, so the *y*-axis scale of Fig. 4 ▸(*b*) is much smaller than that of Fig. 4 ▸(*a*).

Next, we examine the intensity fit in Fig. 4 ▸(*c*). Though the 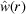 curves in Figs. 4 ▸(*a*) and 4 ▸(*b*) look very different, they all predict similar intensity curves, as shown in Fig. 4 ▸(*c*). Again, this displays the effect of structural ambiguity in SAS inversion. We show in the previous example that, by changing the parameter resolution, *FFSAS* can provide the user with a series of good solutions for further consideration. In this example, we show that the solutions at different trust-region iterations from a single run can also serve this purpose.

### 3.5. SAXS from a bimodal mixture of polydisperse spheres

This SAXS data set was obtained from a dispersion composed of two known calibrants, verified against NIST SRMs 1690 and 1691. The sample was a 50/50 (*v*/*v*) mixture of commercially purchased polystyrene nanoparticles possessing radii of 625 ± 25 and 1025 ± 30 Å, as per their certificates of analysis.

**Figure 5:**
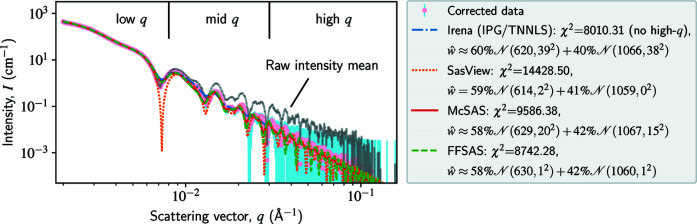
Size-distribution inversion of polydisperse spheres using a SAXS data set. The intensity data contain 1024 points, all used for inversion (except for *Irena*). To account for an experimental artefact, we apply a power-law correction to the mean of the intensity data across the mid-*q* and high-*q* ranges (namely, we use a power-law background); the mean curve before this correction is plotted in grey. The radius *r* ranges from 400 to 1200 Å, discretized by 1000 points. We do not show the inverted 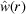 curves here; instead, their bimodal Gaussian approximations are given in the legend. For *SasView*, we assume that the functional form of *w*(*r*) is composed of two Gaussians. To obtain a stable solution from *Irena*, we had to truncate the noisy high-*q* end and switch from MaxEnt to the IPG/TNNLS (interior point gradient/total non-negative least squares) algorithm.

The intensity data and our results are shown in Fig. 5 ▸. For a known experimental reason, the original data suffer from an upward drifting across the mid-*q* and high-*q* ranges; to correct for this artefact, we use a power-law source background instead of a flat one (Ilavsky & Jemian, 2009 ▸). The 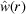 curves found by *Irena*, *SasView*, *McSAS* and *FFSAS* are in good agreement, all identifying two populations centred around 620 and 1060 Å with a volume ratio near 60/40. These numbers are consistent with our prior knowledge of the sample: the inverted radii lie within their certificated ranges and the volume ratio deviates from the truth by less than 10%. However, the 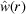 curves from *FFSAS* and *SasView* are highly localized at the two centres, while those from *Irena *and *McSAS* are more dispersive. The localized solutions are more consistent with the truth that the sample only contains two types of uni-size particles. To obtain such localized solutions again requires an accurate inverse solver.

### 3.6. Non-dilute systems of polydisperse spheres

**Figure 6:**
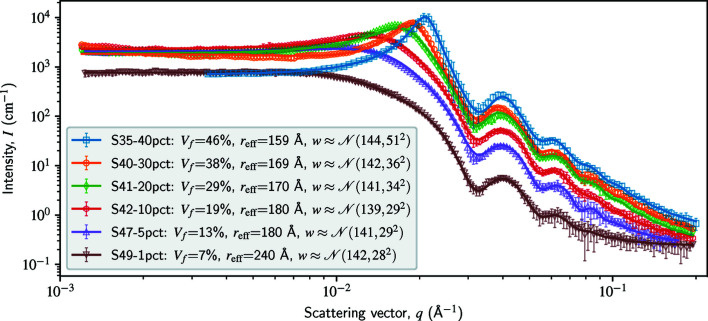
Non-dilute systems of polydisperse spheres from a USAXS data set for LUDOX colloidal silica in a range of dilutions. The intensity curves contain 160–260 points, uniformly distributed between 10^−3^ and 0.2 Å^−1^ in logarithmic scale. Our radius parameter ranges between 1 and 10^2.5^ Å, uniformly discretized by 1000 points in logarithmic scale. We use the hard-sphere structure factor, which includes two variables, the effective radius (*r*_eff_) and the volume fraction (*V*_f_). To handle the non-convexity of the inverse problem, we conduct a brute-force search for their initial guess, considering five effective radii ranging from 100 to 300 Å and seven volume fractions from 1 to 50%. We do not show the inverted *w*(*r*) curves here; instead, their Gaussian approximations are given in the legend.

Our final example demonstrates the inversion of a non-dilute system with a structure factor. We used an ultra-small-angle X-ray scattering (USAXS) data set for LUDOX colloidal silica in a range of dilutions, created as part of the *GSAS-II* package (Toby & Von Dreele, 2013 ▸) for a tutorial (https://subversion.xray.aps.anl.gov/pyGSAS/Tutorials/SAseqref/). Furthermore, we used the ‘hard-sphere’ structure factor. Fig. 6 ▸ shows our results, which are similar to those obtained from *GSAS-II* and *SasView* (both, however, assume an analytical size distribution).

The hard-sphere structure factor introduces two variables to our Green tensor: the effective radius (*r*_eff_) and the volume fraction (*V*_f_). These variables will break the convexity of the inverse problem, making the solution dependent on the initial guess of the two variables. In the *GSAS-II* tutorial, this difficulty is tackled by hand-tuning the initial guess utilizing a GUI; here we conduct a brute-force search over a coarse grid for five effective radii and seven volume fractions – in other words, we try 35 initial guesses. In a future version of *FFSAS*, we will provide the option to use a global optimization algorithm to handle non-convex problems such as this one.

## 4. Conclusions

The method described in this article is developed for free-form parameter inversion of a polydisperse system in SAS. We formulate the forward problem of SAS modelling with polydispersity as a multi-linear map characterized by a high-dimensional Green tensor. The inverse problem then emerges as a constrained NLP targeted at the MLE of the model parameters. Our forward and inverse formulation is general enough to consider (1) any theoretical model with multiple polydisperse parameters, (2) 1D and 2D scattering intensity observations, and (3) any physical or experimental effects that can be built into the Green tensor (such as the structure factors and resolution functions). We solve the inverse problem with high accuracy and efficiency based on several theoretical and computational enhancements, such as accuracy preservation via an automatic data scaling and GPU-accelerated chunk computation for large-scale problems.

Our method is implemented as a Python library called *FFSAS*. Our numerical examples show two advantages of *FFSAS* compared with the existing codes we have tested. First, its ultra-high accuracy allows it to deliver solutions in an overfitting regime, which cannot be found by any of the previous methods (we will elaborate this in the following subsection). Second, thanks to its high computational performance, it can efficiently solve large-scale multi-parameter problems in free form; among the compared codes, only *McSAS* can solve problems of this kind, which is, however, slower than *FFSAS* by at least one to two orders of magnitude.

### 4.1. Structural ambiguity

As shown by our numerical examples, SAS inversion is ill-posed, subject to a high degree of non-uniqueness of solutions or structural ambiguity. The neighbourhood of the MLE is convex but nearly flat, from which the different-looking parameter distributions can predict an ‘identical’ scattering intensity as measured in reference to data uncertainty. An estimator closer to the MLE (or giving a smaller fitting error) may not necessarily be more physically plausible due to overfitting of the noise. Regularizing the fitting error with some additional constraints (such as MaxEnt) can provide a means of solution selection, which, however, is also subjective and non-physical. As we recommend, the most reliable way of handling structural ambiguity is to provide a series of solutions that fit the data to different acceptable levels, across the transition from underfitting to overfitting, from which the user can select one based on other physical or empirical considerations.

To approach the overfitting regime, however, the inverse solver needs to be highly accurate to minimize the fitting error for more significant digits. The lower the noise level is, the more accurate the inverse solver needs to be. For example, at the limit of a noise-free intensity observation, the inverse solver must be able to reduce the fitting error to a machine-epsilon level. In light of the continuous effort to improve SAS experimentation for higher-quality observations, developing more accurate methods for SAS data analysis should also become increasingly important.

Based on our algorithmic enhancements, *FFSAS* proves to be sufficiently accurate to approach the overfitting regime, while the other form-free methods we have tested mostly work in an underfitting regime. For instance, in Fig. 4 ▸, *FFSAS* can deliver a series of solutions from dispersive (underfitting) to localized (overfitting) for a single run, while the other form-free methods can only yield a dispersive one. 

## Figures and Tables

**Figure 7:**
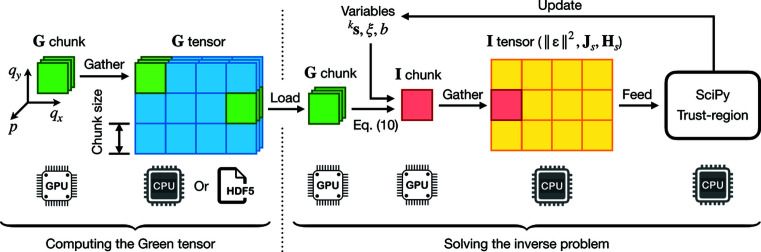
Architecture of GPU-accelerated chunk computation for large-scale multi-parameter problems. In this figure, we assume *M* = 2 and denote the two scattering vectors by *q*_*x*_ and *q*_*y*_. Chunking is performed along these two dimensions. All the model-parameter dimensions are conceptually represented by the *p* dimension. Left: given a SAS model and its parameter space, we compute **G** in chunks on a GPU and store it on disk if needed. Right: to compute any term in 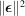 , **J**_*s*_ or **H**_*s*_ that requires successive inner products with **G**, we chunk it along the *q* dimensions and load the corresponding chunk of **G** on a GPU to perform the inner products; the assembled results are then fed to the trust-region method to update the variables.

**Table 1:** Fitting errors and computational cost of solutions given by the four codes The benchmark problem and the prerequisites for some of the solutions are described in Fig. 1 ▸. The wt values were measured on a CPU.

Method	χ^2^	*∥*Δ**w** *∥*	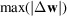	wt (s)
*Irena* (MaxEnt)	7 × 10^−1^	6 × 10^−3^	2 × 10^−3^	2
*SasView*	2 × 10^−3^	4 × 10^−4^	8 × 10^−5^	0.1
*McSAS*	2 × 10^−4^	7 × 10^−3^	2 × 10^−3^	200
*FFSAS* (ours)	9 × 10^−13^	4 × 10^−4^	2 × 10^−4^	3

## Appendix A Solving NLP-w

Solving NLP-w, equations (5*a*)–(5*c*) [Disp-formula fd7], is not straightforward. It is an ill-posed large-scale NLP with mixed equality and inequality constraints. In this appendix, we introduce several techniques that make NLP-w solvable with high accuracy and efficiency.

### A1. Elimination of inequality constraints

The first difficulty we must overcome is that equation (5*b*) contains  inequality constraints, significantly slowing down the solution for a high-resolution or multi-parameter problem. This is because the state-of-the-art NLP solvers are still not highly efficient in handling a large number of inequality constraints. Here we eliminate the inequality constraints by introducing a slack variable ^*k*^ **s**, such that  , turning NLP-w into the following NLP named NLP-s:E

subject to

with equation (1) reformed as a function of ^*k*^ **s**,

Containing only *N* equality constraints, NLP-s has much lower algorithmic complexity than NLP-w, even with the polynomial order of  increased from quadratic to quartic.

### A2. Accuracy preservation

In NLP, the orders of magnitude of the variables cannot vary too drastically; otherwise, the Hessian of the objective function will become ill conditioned, leading to inaccurate or incorrect results. In NLP-w, the ^*k*^ **w** values are dimensionless, ranging between 0 and 1, while *b* and ξ have the base units of intensity and intensity divided by the Green tensor, respectively. One can easily show that the base units of ξ and *b* differ by m^6^, and their numerical values can differ by up to 10^20^ for a typical neutron or X-ray data set using a length unit near nanometres. To avoid this large gap, one workaround is to handcraft a unit convention based on typical use cases, such as the one adopted by *SasView *(Doucet *et al.*, 2021 ▸) and many other codes. This is, however, inflexible and may still fail for a non-typical application.

For the inverse problem, we aim to preserve the numerical accuracy of forward modelling given any unit system of the input data (  ,  and **G**). The idea is to find an intermediate unit system under which ξ and *b* become dimensionless and numerically close to 1. Clearly, such an intermediate unit system must be a function of  ,  and **G**. Let us assume that all the parameter distributions are uniform, *i.e.* ^*k*^ *w*_*i*_ = 1/*n*_*k*_. Under this assumption, NLP-w degenerates to a standard quadratic problem of ξ and *b*. Let (ξ_0_, *b*_0_) be the minimizer of this quadratic NLP, which should be a good approximation to the real minimizer of NLP-w as measured by their orders of magnitude. Therefore, we can make ξ and *b* dimensionless and close to 1 by using *b*_0_ as the new unit for intensity and *b*_0_/ξ_0_ as that for the Green tensor. In summary, we feed  ,  and **G**ξ_0_/*b*_0_ into NLP-s to solve variables ^*k*^ **s**, ξ/ξ_0_ and *b*/*b*_0_. The closed-form expressions for ξ_0_ and *b*_0_ can be easily shown as

where

 is the mean of **G** along the parameter ranks,

Note that ^*k*^ *w*_*i*_ = 1/*n*_*k*_, ξ = ξ_0_ and *b* = *b*_0_ also make a good initial guess for NLP-w.

### A3. Trust-region method

We solve the inverse problem NLP-s using the Byrd–Omojokun trust-region method (Lalee *et al.*, 1998 ▸) implemented in *SciPy* (Virtanen *et al.*, 2020 ▸). According to the *SciPy* documentation, ‘it is the most versatile constrained minimization algorithm implemented in *SciPy* and the most appropriate for large-scale problems’. Using the nonlinear conjugate-gradient method as the underlying solver for unconstrained NLP, the trust-region method demands the Jacobian and Hessian of  with respect to {^*k*^ **s**, ξ, *b*}, as denoted by **J**_*s*_ and **H**_*s*_, respectively. Using equations (4) and (10), the closed-form expressions of **J**_*s*_ and **H**_*s*_ can be derived, which can significantly speed up the solution process compared with computing them by finite difference. Because  is a quartic function of ^*k*^ **s**, these closed-form expressions are lengthy and omitted from the article.

Two computational challenges remain. First, the size of the Green tensor **G** can grow exceedingly large for a multi-parameter model; for example, given a model with *M* = 2 and *N* = 4, and ^1^ *q*, ^2^ *q*, ^1^ *p*, ^2^ *p*, ^3^ *p* and ^4^ *p* all discretized by 50 points, **G** has 50^6^ elements, requiring 125 GB of memory in double-precision floats. Second, the trust-region solver needs to calculate  , **J**_*s*_ and **H**_*s*_ hundreds of times in one inversion; despite their closed-form expressions, such calculations can still be computationally expensive owing to the successive inner products in equation (10). We overcome these two difficulties using the strategy of GPU-accelerated chunk computation, based on the deep-learning library *PyTorch* (Paszke *et al.*, 2019 ▸). Our computational architecture is elaborated in Fig. 7 ▸. A GPU is needed only for large-scale multi-parameter problems; for a low-dimensional problem, such as size-distribution inversion of polydisperse spheres (*N* = 1), even at an ultra-high resolution, our runtime is usually a few seconds on a CPU.

### A4. On-the-fly dimension reduction

As governed by the successive inner products in equations (1) or (10), the algorithmic complexity of the inverse problem is bounded by  . Even with the GPU-accelerated chunk computation, the solution can still be time consuming for a multi-parameter model with a large parameter space. In view of the multiplication  , the runtime can be significantly decreased if one or some of the parameter dimensions can be reduced on the fly. For most multi-parameter SAS models, such dimension reduction is theoretically permitted because their intensity function should be more sensitive to some of the parameters than to others, and these parameters will converge quicker during the trust-region iterations. For example, considering polydisperse cylinders with randomly oriented axes, the radius distribution will converge much faster than the length distribution because the volume of a cylinder (and thus the scattering amplitude) scales with length but with radius squared. All we need to do is to monitor the convergence of each parameter distribution after each trust-region iteration, marking any converged parameters as constants for further iterations.
